# It is a real hazelnit allergy? The CCD interference

**DOI:** 10.1186/2045-7022-1-S1-P71

**Published:** 2011-08-12

**Authors:** Antonio Nicola Romeo, Giuseppe Menna

**Affiliations:** 1 ISS -State Hospital - San Marino Republic (RSM), Pediatrics, Borgo Maggiore, San Marino

## Bacground

Cross-reactive carbohydrate determinants (CCD) are carbohydrates chains in glycoproteins. Capable of binding human IgE from allergic they could play a role in the cross-reactivity between allergens from unrelated sources; their function still a matter of debate.

## Objective

Pollen is the most important cause of the production of IgE anti-CCD. Which have been described in trees and herbs. We investigated the relationship between clinical anaphylaxis to hazelnut and the sierological positivity to nuts allergen, grasses and role of interference by anti-CCD IgE.

## Methods

A six year old twins, atopic risk, father suffering from pollen asthma, reported by parents adverse reaction (Sampson, 2003 1st -2 nd grade) to hazelnut at first assumption.

## Results

Skin tests positive for nuts and grasses, no mite high RAST positivity for grasses, over 100 KUI/L, Alternaria 34 KUI/L, Phl-1 83,1KUI/L, Alt a-1- 43KUI/L, Cor a8 negative, Nuts negative, MUFXF3 ( CCD) 2.83 U/ml. Food Challenge after 1 year: negative, Now allergic rhinithis.

## Conclusion

IgE anti-CCD should not be able to connect mast cells and basophils and release inflammatory mediators. Patients with IgE restricted to CCD have not clinical symptoms. CCD can be considered as a potential interference in atopic diagnosis; especially in polisensitized. It 's important to provide clinical information to laboratory test. IgE -anti CCD create a misdiagnosis of allergies to foods, latex or venom, especially in pollinosis This can lead to unnecessary therapy.
